# 3-(1*H*-Benzimidazol-2-yl)-2-chloro-8-methyl­quinoline

**DOI:** 10.1107/S1600536809002827

**Published:** 2009-01-28

**Authors:** Frank Rominger, Mahalingam Malathi, Palathurai Subramaniam Mohan, Tanuja Ramamurthi Dondeti, A. Stephen K. Hashmi

**Affiliations:** a Organisch-Chemisches Institut, Universität Heidelberg, Im Neuenheimer Feld 270, 69120 Heidelberg, Germany; b Department of Chemistry, Bharathiar University, Coimbatore 641 046, India

## Abstract

Two independent mol­ecules of the title compound, C_17_H_12_ClN_3_, are present in the structure. The angle between the planes defined by the atoms of the benzimidazole unit and the quinoline unit are 45.2 (3) and 44.0 (3)°, indicating an essentially identical conformation for both mol­ecules. Each of the independent mol­ecules is linked with a symmetry equivalent by an inter­molecular N—H⋯N hydrogen bond involving the two benzimidazole N atoms, to form chains in the crystallographic *c* direction.

## Related literature

A closely related structure is reported by Rominger *et al.* (2009 ▶). An analogous pyridine compound is essentially flat (Kim *et al.*, 2005 ▶).
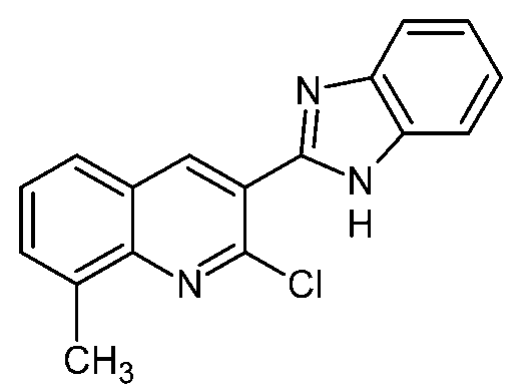


## Experimental

### 

#### Crystal data

C_17_H_12_ClN_3_
*M* *_r_* = 293.75
Monoclinic, 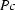

*a* = 16.4721 (15) Å
*b* = 9.0061 (8) Å
*c* = 9.6643 (9) Å
β = 98.433 (2)°
*V* = 1418.2 (2) Å^3^
*Z* = 4
Mo *K*α radiation
μ = 0.27 mm^−1^
*T* = 200 (2) K
0.41 × 0.16 × 0.11 mm

#### Data collection

Bruker SMART APEX diffractometer
Absorption correction: multi-scan (**SADABS**; Sheldrick, 2008*b* ▶) *T*_min_ = 0.897, *T*_max_ = 0.969
14446 measured reflections
6767 independent reflections
6457 reflections with *I* > 2σ(*I*)
*R*_int_ = 0.025

#### Refinement

*R*[*F* ^2^ > 2σ(*F* ^2^)] = 0.042
*wR*(*F* ^2^) = 0.100
*S* = 1.12
6767 reflections
381 parameters
2 restraints
H-atom parameters constrained
Δρ_max_ = 0.33 e Å^−3^
Δρ_min_ = −0.22 e Å^−3^
Absolute structure: Flack (1983 ▶), 3244 Friedel pairs
Flack parameter: −0.03 (5)

### 

Data collection: *SMART* (Bruker, 2001 ▶); cell refinement: *SAINT* (Bruker, 2001 ▶); data reduction: *SAINT*; program(s) used to solve structure: *SHELXTL* (Sheldrick, 2008*a* ▶); program(s) used to refine structure: *SHELXTL*; molecular graphics: *SHELXTL*; software used to prepare material for publication: *SHELXTL*.

## Supplementary Material

Crystal structure: contains datablocks I, global. DOI: 10.1107/S1600536809002827/fj2183sup1.cif

Structure factors: contains datablocks I. DOI: 10.1107/S1600536809002827/fj2183Isup2.hkl

Additional supplementary materials:  crystallographic information; 3D view; checkCIF report

**Fig. 3.**
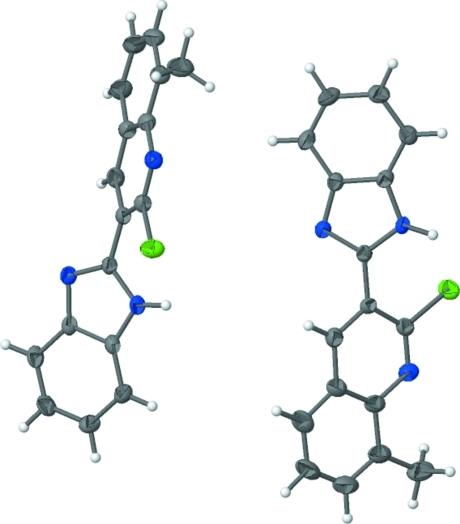
Enhanced figure of both independent molecules of the title compound with displacement ellipsoids plotted at 50% probability level.

Enhanced figure: interactive version of Fig. 3

## Figures and Tables

**Table 1:** Hydrogen-bond geometry (Å, °)

*D*—H⋯*A*	*D*—H	H⋯*A*	*D*⋯*A*	*D*—H⋯*A*
N14—H14⋯N13^i^	0.88	2.05 (1)	2.851 (2)	150
N14*B*—H14*B*⋯N13*B*^ii^	0.88	2.02 (1)	2.826 (2)	151

Symmetry codes: (i) 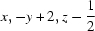; (ii) 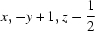.

**Fig. 1.**
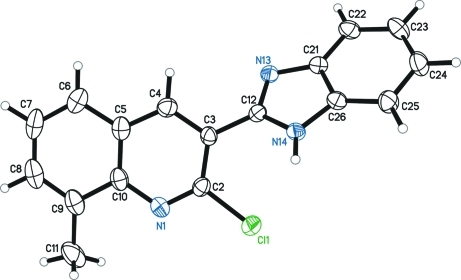
Thermal ellipsoid representation of the title compound with displacement ellipsoids plotted at 50% probability level. Only one of the two independent molecules is shown.

**Fig. 2.**
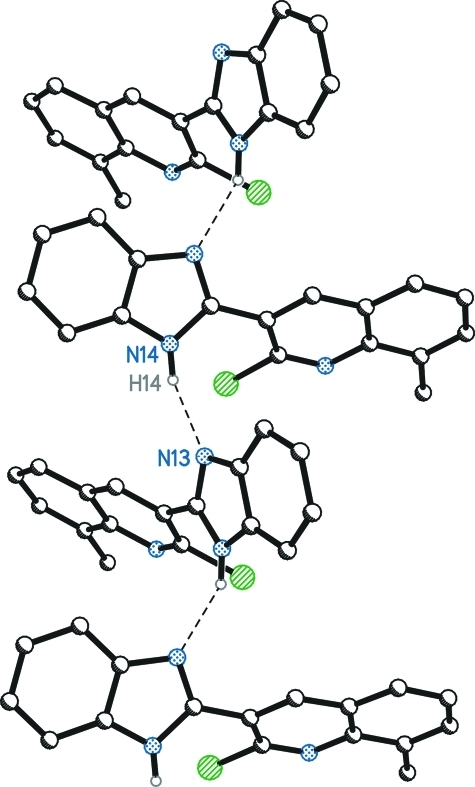
Ball and stick representation of the hydrogen bond connected chain along the c direction. Hydrogen atoms not involved in hydrogen bonds (dashed lines) have been omitted. Each of the two independent molecules forms such an assembly, the analogous chain of the second molecule is not depicted.

## supplementary crystallographic information

### Refinement

For all hydrogen atoms the positions were calculated according to geometrical criteria. Planar geometry was assumed for the nitrogen atom of the benzimidazol unit. During the refinement the hydrogen atoms were allowed to shift with the preceding atoms. In the case of the methyl groups the torsion angles were allowed to refine. The isotropic displacement parameters were set as 1.2 times (1.5 for methyl) the equivalent isotropic displacement parameters of the preceding atoms.

### Crystal data

**Table:**

C_17_H_12_ClN_3_	*F*(000) = 608
*M**_r_* = 293.75	*D*_x_ = 1.376 Mg m^−^^3^
Monoclinic, *P**c*	Mo *K*α radiation, λ = 0.71073 Å
Hall symbol: P -2yc	Cell parameters from 5811 reflections
*a* = 16.4721 (15) Å	θ = 2.5–28.2°
*b* = 9.0061 (8) Å	µ = 0.27 mm^−^^1^
*c* = 9.6643 (9) Å	*T* = 200 K
β = 98.433 (2)°	Polyhedron, colourless
*V* = 1418.2 (2) Å^3^	0.41 × 0.16 × 0.11 mm
*Z* = 4	

### Data collection

**Table:**

Bruker APEX diffractometer	6767 independent reflections
Radiation source: fine-focus sealed tube	6457 reflections with *I* > 2σ(*I*)
graphite	*R*_int_ = 0.025
ω scans	θ_max_ = 28.3°, θ_min_ = 2.3°
Absorption correction: multi-scan (*SADABS*; Sheldrick, 2008b)	*h* = −21→21
*T*_min_ = 0.897, *T*_max_ = 0.969	*k* = −11→12
14446 measured reflections	*l* = −12→12

### Refinement

**Table:**

Refinement on *F*^2^	Secondary atom site location: difference Fourier map
Least-squares matrix: full	Hydrogen site location: inferred from neighbouring sites
*R*[*F*^2^ > 2σ(*F*^2^)] = 0.042	H-atom parameters constrained
*wR*(*F*^2^) = 0.100	*w* = 1/[σ^2^(*F*_o_^2^) + (0.0479*P*)^2^ + 0.2051*P*] where *P* = (*F*_o_^2^ + 2*F*_c_^2^)/3
*S* = 1.12	(Δ/σ)_max_ = 0.001
6767 reflections	Δρ_max_ = 0.33 e Å^−^^3^
381 parameters	Δρ_min_ = −0.22 e Å^−^^3^
2 restraints	Absolute structure: Flack (1983), 3233 Friedel pairs
Primary atom site location: structure-invariant direct methods	Flack parameter: −0.03 (5)

### Special details

**Table:**

Geometry. All e.s.d.'s (except the e.s.d. in the dihedral angle between two l.s. planes) are estimated using the full covariance matrix. The cell e.s.d.'s are taken into account individually in the estimation of e.s.d.'s in distances, angles and torsion angles; correlations between e.s.d.'s in cell parameters are only used when they are defined by crystal symmetry. An approximate (isotropic) treatment of cell e.s.d.'s is used for estimating e.s.d.'s involving l.s. planes.
Refinement. Refinement of *F*^2^ against ALL reflections. The weighted *R*-factor *wR* and goodness of fit *S* are based on *F*^2^, conventional *R*-factors *R* are based on *F*, with *F* set to zero for negative *F*^2^. The threshold expression of *F*^2^ > σ(*F*^2^) is used only for calculating *R*-factors(gt) *etc*. and is not relevant to the choice of reflections for refinement. *R*-factors based on *F*^2^ are statistically about twice as large as those based on *F*, and *R*- factors based on ALL data will be even larger.

### Fractional atomic coordinates and isotropic or equivalent isotropic displacement parameters (Å^2^)

**Table:**

	*x*	*y*	*z*	*U*_iso_*/*U*_eq_	
Cl1	0.05087 (4)	1.03625 (6)	−0.15249 (6)	0.03642 (14)	
N1	0.02863 (11)	0.7608 (2)	−0.1012 (2)	0.0295 (4)	
C2	0.07851 (13)	0.8696 (2)	−0.0672 (2)	0.0263 (4)	
C3	0.15072 (13)	0.8633 (2)	0.0341 (2)	0.0244 (4)	
C4	0.16618 (14)	0.7291 (2)	0.0999 (2)	0.0306 (5)	
H4	0.2127	0.7190	0.1700	0.037*	
C5	0.11470 (15)	0.6067 (2)	0.0658 (2)	0.0322 (5)	
C6	0.12919 (17)	0.4661 (3)	0.1294 (3)	0.0405 (6)	
H6	0.1754	0.4508	0.1991	0.049*	
C7	0.0760 (2)	0.3524 (3)	0.0897 (3)	0.0472 (7)	
H7	0.0860	0.2571	0.1308	0.057*	
C8	0.00720 (19)	0.3748 (3)	−0.0107 (3)	0.0457 (6)	
H8	−0.0292	0.2940	−0.0344	0.055*	
C9	−0.01022 (16)	0.5079 (3)	−0.0765 (3)	0.0382 (5)	
C10	0.04522 (14)	0.6272 (2)	−0.0366 (2)	0.0304 (5)	
C11	−0.08315 (19)	0.5301 (4)	−0.1860 (3)	0.0526 (8)	
H11A	−0.0655	0.5316	−0.2786	0.079*	
H11B	−0.1097	0.6246	−0.1697	0.079*	
H11C	−0.1221	0.4485	−0.1817	0.079*	
C12	0.20363 (13)	0.9916 (2)	0.0744 (2)	0.0230 (4)	
N13	0.23031 (11)	1.02682 (19)	0.20545 (18)	0.0253 (4)	
N14	0.22948 (11)	1.08601 (19)	−0.01916 (17)	0.0248 (3)	
H14	0.2184	1.0802	−0.1109	0.030*	
C21	0.27661 (12)	1.1546 (2)	0.1966 (2)	0.0239 (4)	
C22	0.31876 (14)	1.2431 (3)	0.3023 (2)	0.0313 (5)	
H22	0.3191	1.2194	0.3982	0.038*	
C23	0.35963 (15)	1.3654 (3)	0.2630 (2)	0.0368 (5)	
H23	0.3896	1.4259	0.3332	0.044*	
C24	0.35834 (15)	1.4032 (3)	0.1227 (3)	0.0364 (5)	
H24	0.3869	1.4891	0.0995	0.044*	
C25	0.31608 (15)	1.3176 (3)	0.0163 (2)	0.0327 (5)	
H25	0.3144	1.3434	−0.0794	0.039*	
C26	0.27646 (13)	1.1928 (2)	0.0567 (2)	0.0252 (4)	
Cl1B	0.75134 (3)	0.47673 (6)	0.52013 (5)	0.03402 (13)	
N1B	0.77153 (12)	0.7516 (2)	0.58632 (19)	0.0286 (4)	
C2B	0.72268 (13)	0.6411 (2)	0.5955 (2)	0.0254 (4)	
C3B	0.65160 (13)	0.6429 (2)	0.6622 (2)	0.0244 (4)	
C4B	0.63496 (14)	0.7753 (2)	0.7222 (2)	0.0298 (5)	
H4B	0.5887	0.7825	0.7701	0.036*	
C5B	0.68484 (14)	0.9000 (2)	0.7145 (2)	0.0292 (4)	
C6B	0.66884 (16)	1.0405 (3)	0.7716 (3)	0.0378 (5)	
H6B	0.6231	1.0531	0.8199	0.045*	
C7B	0.71925 (17)	1.1565 (3)	0.7568 (3)	0.0405 (6)	
H7B	0.7082	1.2509	0.7937	0.049*	
C8B	0.78792 (16)	1.1379 (3)	0.6871 (3)	0.0393 (6)	
H8B	0.8227	1.2208	0.6794	0.047*	
C9B	0.80661 (15)	1.0060 (3)	0.6302 (2)	0.0341 (5)	
C10B	0.75394 (13)	0.8840 (2)	0.6439 (2)	0.0281 (4)	
C11B	0.87946 (19)	0.9877 (3)	0.5563 (3)	0.0473 (7)	
H11D	0.9076	1.0833	0.5534	0.071*	
H11E	0.8615	0.9529	0.4607	0.071*	
H11F	0.9172	0.9150	0.6063	0.071*	
C12B	0.59921 (13)	0.5128 (2)	0.6756 (2)	0.0238 (4)	
N13B	0.57193 (11)	0.4767 (2)	0.79249 (18)	0.0252 (4)	
N14B	0.57387 (11)	0.4184 (2)	0.56856 (17)	0.0271 (4)	
H14B	0.5853	0.4246	0.4826	0.033*	
C21B	0.52618 (12)	0.3494 (2)	0.7604 (2)	0.0243 (4)	
C22B	0.48330 (14)	0.2607 (3)	0.8442 (2)	0.0318 (5)	
H22B	0.4824	0.2840	0.9398	0.038*	
C23B	0.44264 (15)	0.1387 (3)	0.7833 (2)	0.0365 (5)	
H23B	0.4125	0.0779	0.8378	0.044*	
C24B	0.44435 (15)	0.1015 (3)	0.6433 (3)	0.0367 (5)	
H24B	0.4157	0.0158	0.6052	0.044*	
C25B	0.48679 (15)	0.1867 (3)	0.5596 (2)	0.0330 (5)	
H25B	0.4885	0.1613	0.4646	0.040*	
C26B	0.52677 (12)	0.3110 (2)	0.6203 (2)	0.0245 (4)	

### Atomic displacement parameters (Å^2^)

**Table:**

	*U*^11^	*U*^22^	*U*^33^	*U*^12^	*U*^13^	*U*^23^
Cl1	0.0340 (3)	0.0292 (3)	0.0432 (3)	0.0011 (2)	−0.0042 (2)	0.0066 (2)
N1	0.0286 (9)	0.0294 (9)	0.0306 (9)	−0.0025 (7)	0.0045 (7)	−0.0048 (7)
C2	0.0310 (11)	0.0228 (10)	0.0258 (10)	0.0034 (8)	0.0062 (8)	0.0004 (8)
C3	0.0313 (10)	0.0211 (9)	0.0213 (9)	−0.0013 (8)	0.0052 (8)	−0.0030 (7)
C4	0.0357 (12)	0.0312 (11)	0.0246 (10)	0.0010 (9)	0.0033 (8)	0.0006 (8)
C5	0.0395 (12)	0.0267 (11)	0.0328 (11)	−0.0030 (9)	0.0138 (9)	−0.0024 (9)
C6	0.0486 (15)	0.0300 (13)	0.0444 (14)	0.0068 (11)	0.0120 (11)	0.0056 (10)
C7	0.0707 (19)	0.0222 (12)	0.0532 (16)	−0.0010 (11)	0.0243 (14)	0.0028 (11)
C8	0.0578 (16)	0.0326 (13)	0.0508 (15)	−0.0153 (12)	0.0221 (13)	−0.0107 (11)
C9	0.0431 (13)	0.0353 (12)	0.0390 (13)	−0.0124 (10)	0.0156 (10)	−0.0085 (10)
C10	0.0344 (11)	0.0274 (11)	0.0319 (11)	−0.0047 (9)	0.0125 (9)	−0.0061 (8)
C11	0.0430 (16)	0.0574 (19)	0.0562 (18)	−0.0256 (14)	0.0031 (13)	−0.0075 (14)
C12	0.0273 (10)	0.0216 (9)	0.0202 (9)	0.0031 (8)	0.0040 (7)	−0.0003 (7)
N13	0.0298 (9)	0.0250 (9)	0.0205 (8)	−0.0011 (7)	0.0013 (7)	−0.0003 (6)
N14	0.0307 (9)	0.0277 (9)	0.0157 (7)	−0.0041 (7)	0.0021 (6)	−0.0008 (6)
C21	0.0226 (9)	0.0272 (10)	0.0222 (9)	0.0005 (8)	0.0038 (7)	−0.0001 (8)
C22	0.0330 (11)	0.0388 (12)	0.0213 (9)	−0.0025 (9)	0.0016 (8)	−0.0040 (9)
C23	0.0348 (12)	0.0401 (13)	0.0343 (12)	−0.0098 (10)	0.0009 (9)	−0.0126 (10)
C24	0.0353 (12)	0.0366 (13)	0.0382 (12)	−0.0129 (10)	0.0086 (9)	−0.0012 (10)
C25	0.0339 (11)	0.0357 (12)	0.0294 (11)	−0.0067 (9)	0.0086 (9)	0.0023 (9)
C26	0.0254 (10)	0.0288 (11)	0.0215 (9)	−0.0002 (8)	0.0037 (7)	−0.0035 (8)
Cl1B	0.0340 (3)	0.0259 (2)	0.0441 (3)	0.0020 (2)	0.0123 (2)	−0.0069 (2)
N1B	0.0301 (9)	0.0269 (9)	0.0284 (9)	0.0002 (7)	0.0026 (7)	0.0008 (7)
C2B	0.0304 (10)	0.0214 (10)	0.0242 (9)	0.0058 (8)	0.0039 (8)	−0.0009 (7)
C3B	0.0300 (10)	0.0236 (10)	0.0196 (9)	0.0002 (8)	0.0040 (7)	0.0019 (7)
C4B	0.0347 (12)	0.0309 (11)	0.0256 (10)	0.0028 (9)	0.0099 (8)	0.0001 (8)
C5B	0.0370 (11)	0.0237 (10)	0.0260 (10)	0.0014 (9)	0.0013 (8)	0.0003 (8)
C6B	0.0456 (14)	0.0288 (12)	0.0383 (13)	0.0069 (10)	0.0042 (10)	−0.0060 (9)
C7B	0.0547 (15)	0.0223 (11)	0.0416 (13)	0.0069 (10)	−0.0028 (11)	−0.0064 (10)
C8B	0.0453 (14)	0.0265 (11)	0.0429 (13)	−0.0060 (10)	−0.0049 (11)	0.0018 (10)
C9B	0.0358 (12)	0.0280 (11)	0.0358 (12)	−0.0040 (9)	−0.0035 (9)	0.0028 (9)
C10B	0.0308 (11)	0.0238 (10)	0.0279 (10)	0.0012 (8)	−0.0020 (8)	0.0009 (8)
C11B	0.0461 (16)	0.0393 (15)	0.0580 (18)	−0.0152 (12)	0.0130 (13)	−0.0037 (12)
C12B	0.0283 (10)	0.0241 (10)	0.0191 (9)	0.0026 (8)	0.0041 (7)	0.0003 (7)
N13B	0.0277 (9)	0.0272 (9)	0.0214 (8)	0.0012 (7)	0.0065 (7)	−0.0007 (6)
N14B	0.0341 (9)	0.0297 (9)	0.0189 (8)	−0.0020 (8)	0.0087 (7)	0.0006 (7)
C21B	0.0237 (9)	0.0289 (10)	0.0204 (9)	0.0035 (8)	0.0040 (7)	0.0010 (8)
C22B	0.0307 (11)	0.0419 (13)	0.0242 (10)	−0.0012 (9)	0.0087 (8)	0.0054 (9)
C23B	0.0347 (12)	0.0416 (13)	0.0344 (12)	−0.0092 (10)	0.0095 (9)	0.0099 (10)
C24B	0.0369 (12)	0.0366 (13)	0.0356 (12)	−0.0118 (10)	0.0024 (9)	−0.0035 (10)
C25B	0.0360 (12)	0.0370 (12)	0.0253 (10)	−0.0065 (10)	0.0013 (9)	−0.0033 (9)
C26B	0.0258 (10)	0.0277 (10)	0.0202 (9)	0.0018 (8)	0.0044 (7)	0.0041 (8)

### Geometric parameters (Å, °)

**Table:**

Cl1—C2	1.740 (2)	Cl1B—C2B	1.745 (2)
N1—C2	1.290 (3)	N1B—C2B	1.291 (3)
N1—C10	1.365 (3)	N1B—C10B	1.365 (3)
C2—C3	1.426 (3)	C2B—C3B	1.417 (3)
C3—C4	1.373 (3)	C3B—C4B	1.371 (3)
C3—C12	1.465 (3)	C3B—C12B	1.472 (3)
C4—C5	1.400 (3)	C4B—C5B	1.400 (3)
C4—H4	0.9500	C4B—H4B	0.9500
C5—C10	1.411 (3)	C5B—C10B	1.418 (3)
C5—C6	1.413 (3)	C5B—C6B	1.421 (3)
C6—C7	1.366 (4)	C6B—C7B	1.355 (4)
C6—H6	0.9500	C6B—H6B	0.9500
C7—C8	1.394 (4)	C7B—C8B	1.409 (4)
C7—H7	0.9500	C7B—H7B	0.9500
C8—C9	1.367 (4)	C8B—C9B	1.363 (3)
C8—H8	0.9500	C8B—H8B	0.9500
C9—C10	1.426 (3)	C9B—C10B	1.418 (3)
C9—C11	1.493 (4)	C9B—C11B	1.493 (4)
C11—H11A	0.9800	C11B—H11D	0.9800
C11—H11B	0.9800	C11B—H11E	0.9800
C11—H11C	0.9800	C11B—H11F	0.9800
C12—N13	1.317 (3)	C12B—N13B	1.316 (3)
C12—N14	1.355 (3)	C12B—N14B	1.357 (3)
N13—C21	1.390 (3)	N13B—C21B	1.382 (3)
N14—C26	1.377 (3)	N14B—C26B	1.379 (3)
N14—H14	0.8800	N14B—H14B	0.8800
C21—C26	1.394 (3)	C21B—C26B	1.399 (3)
C21—C22	1.397 (3)	C21B—C22B	1.400 (3)
C22—C23	1.373 (3)	C22B—C23B	1.373 (3)
C22—H22	0.9500	C22B—H22B	0.9500
C23—C24	1.395 (3)	C23B—C24B	1.398 (3)
C23—H23	0.9500	C23B—H23B	0.9500
C24—C25	1.387 (3)	C24B—C25B	1.378 (3)
C24—H24	0.9500	C24B—H24B	0.9500
C25—C26	1.385 (3)	C25B—C26B	1.385 (3)
C25—H25	0.9500	C25B—H25B	0.9500
			
C2—N1—C10	118.41 (19)	C2B—N1B—C10B	118.30 (19)
N1—C2—C3	125.5 (2)	N1B—C2B—C3B	125.97 (19)
N1—C2—Cl1	114.95 (16)	N1B—C2B—Cl1B	114.57 (16)
C3—C2—Cl1	119.54 (16)	C3B—C2B—Cl1B	119.44 (16)
C4—C3—C2	115.61 (19)	C4B—C3B—C2B	115.62 (19)
C4—C3—C12	120.56 (19)	C4B—C3B—C12B	119.88 (19)
C2—C3—C12	123.73 (18)	C2B—C3B—C12B	124.42 (19)
C3—C4—C5	121.3 (2)	C3B—C4B—C5B	121.3 (2)
C3—C4—H4	119.3	C3B—C4B—H4B	119.4
C5—C4—H4	119.3	C5B—C4B—H4B	119.4
C4—C5—C10	117.6 (2)	C4B—C5B—C10B	117.60 (19)
C4—C5—C6	123.1 (2)	C4B—C5B—C6B	123.4 (2)
C10—C5—C6	119.4 (2)	C10B—C5B—C6B	119.0 (2)
C7—C6—C5	119.3 (3)	C7B—C6B—C5B	119.6 (2)
C7—C6—H6	120.4	C7B—C6B—H6B	120.2
C5—C6—H6	120.4	C5B—C6B—H6B	120.2
C6—C7—C8	120.6 (2)	C6B—C7B—C8B	120.4 (2)
C6—C7—H7	119.7	C6B—C7B—H7B	119.8
C8—C7—H7	119.7	C8B—C7B—H7B	119.8
C9—C8—C7	123.0 (2)	C9B—C8B—C7B	122.8 (2)
C9—C8—H8	118.5	C9B—C8B—H8B	118.6
C7—C8—H8	118.5	C7B—C8B—H8B	118.6
C8—C9—C10	116.9 (2)	C8B—C9B—C10B	117.4 (2)
C8—C9—C11	122.6 (2)	C8B—C9B—C11B	122.3 (2)
C10—C9—C11	120.5 (2)	C10B—C9B—C11B	120.3 (2)
N1—C10—C5	121.6 (2)	N1B—C10B—C5B	121.2 (2)
N1—C10—C9	117.6 (2)	N1B—C10B—C9B	118.0 (2)
C5—C10—C9	120.8 (2)	C5B—C10B—C9B	120.8 (2)
C9—C11—H11A	109.5	C9B—C11B—H11D	109.5
C9—C11—H11B	109.5	C9B—C11B—H11E	109.5
H11A—C11—H11B	109.5	H11D—C11B—H11E	109.5
C9—C11—H11C	109.5	C9B—C11B—H11F	109.5
H11A—C11—H11C	109.5	H11D—C11B—H11F	109.5
H11B—C11—H11C	109.5	H11E—C11B—H11F	109.5
N13—C12—N14	113.42 (18)	N13B—C12B—N14B	113.23 (19)
N13—C12—C3	123.21 (19)	N13B—C12B—C3B	123.41 (19)
N14—C12—C3	123.37 (18)	N14B—C12B—C3B	123.36 (18)
C12—N13—C21	104.42 (17)	C12B—N13B—C21B	104.81 (17)
C12—N14—C26	106.86 (17)	C12B—N14B—C26B	106.83 (17)
C12—N14—H14	126.6	C12B—N14B—H14B	126.6
C26—N14—H14	126.6	C26B—N14B—H14B	126.6
N13—C21—C26	109.92 (17)	N13B—C21B—C26B	109.97 (18)
N13—C21—C22	130.18 (19)	N13B—C21B—C22B	130.41 (19)
C26—C21—C22	119.89 (19)	C26B—C21B—C22B	119.6 (2)
C23—C22—C21	117.8 (2)	C23B—C22B—C21B	117.7 (2)
C23—C22—H22	121.1	C23B—C22B—H22B	121.2
C21—C22—H22	121.1	C21B—C22B—H22B	121.2
C22—C23—C24	121.8 (2)	C22B—C23B—C24B	121.9 (2)
C22—C23—H23	119.1	C22B—C23B—H23B	119.0
C24—C23—H23	119.1	C24B—C23B—H23B	119.0
C25—C24—C23	121.3 (2)	C25B—C24B—C23B	121.2 (2)
C25—C24—H24	119.4	C25B—C24B—H24B	119.4
C23—C24—H24	119.4	C23B—C24B—H24B	119.4
C26—C25—C24	116.6 (2)	C24B—C25B—C26B	116.9 (2)
C26—C25—H25	121.7	C24B—C25B—H25B	121.6
C24—C25—H25	121.7	C26B—C25B—H25B	121.6
N14—C26—C25	131.94 (19)	N14B—C26B—C25B	132.18 (19)
N14—C26—C21	105.38 (18)	N14B—C26B—C21B	105.15 (17)
C25—C26—C21	122.66 (19)	C25B—C26B—C21B	122.65 (19)
			
C10—N1—C2—C3	−0.2 (3)	C10B—N1B—C2B—C3B	−0.9 (3)
C10—N1—C2—Cl1	−178.62 (15)	C10B—N1B—C2B—Cl1B	−179.39 (15)
N1—C2—C3—C4	−0.7 (3)	N1B—C2B—C3B—C4B	−0.4 (3)
Cl1—C2—C3—C4	177.67 (16)	Cl1B—C2B—C3B—C4B	178.08 (15)
N1—C2—C3—C12	−177.0 (2)	N1B—C2B—C3B—C12B	−177.3 (2)
Cl1—C2—C3—C12	1.4 (3)	Cl1B—C2B—C3B—C12B	1.2 (3)
C2—C3—C4—C5	1.5 (3)	C2B—C3B—C4B—C5B	1.2 (3)
C12—C3—C4—C5	177.9 (2)	C12B—C3B—C4B—C5B	178.29 (19)
C3—C4—C5—C10	−1.4 (3)	C3B—C4B—C5B—C10B	−0.8 (3)
C3—C4—C5—C6	179.0 (2)	C3B—C4B—C5B—C6B	178.2 (2)
C4—C5—C6—C7	−179.8 (2)	C4B—C5B—C6B—C7B	−178.5 (2)
C10—C5—C6—C7	0.6 (4)	C10B—C5B—C6B—C7B	0.5 (3)
C5—C6—C7—C8	−1.2 (4)	C5B—C6B—C7B—C8B	−0.9 (4)
C6—C7—C8—C9	1.4 (4)	C6B—C7B—C8B—C9B	0.9 (4)
C7—C8—C9—C10	−1.0 (4)	C7B—C8B—C9B—C10B	−0.3 (4)
C7—C8—C9—C11	178.7 (3)	C7B—C8B—C9B—C11B	179.9 (2)
C2—N1—C10—C5	0.3 (3)	C2B—N1B—C10B—C5B	1.3 (3)
C2—N1—C10—C9	−179.4 (2)	C2B—N1B—C10B—C9B	−178.36 (19)
C4—C5—C10—N1	0.5 (3)	C4B—C5B—C10B—N1B	−0.5 (3)
C6—C5—C10—N1	−179.9 (2)	C6B—C5B—C10B—N1B	−179.5 (2)
C4—C5—C10—C9	−179.8 (2)	C4B—C5B—C10B—C9B	179.16 (19)
C6—C5—C10—C9	−0.1 (3)	C6B—C5B—C10B—C9B	0.1 (3)
C8—C9—C10—N1	−179.9 (2)	C8B—C9B—C10B—N1B	179.4 (2)
C11—C9—C10—N1	0.4 (3)	C11B—C9B—C10B—N1B	−0.7 (3)
C8—C9—C10—C5	0.3 (3)	C8B—C9B—C10B—C5B	−0.2 (3)
C11—C9—C10—C5	−179.4 (2)	C11B—C9B—C10B—C5B	179.7 (2)
C4—C3—C12—N13	−43.2 (3)	C4B—C3B—C12B—N13B	−42.0 (3)
C2—C3—C12—N13	132.8 (2)	C2B—C3B—C12B—N13B	134.8 (2)
C4—C3—C12—N14	137.2 (2)	C4B—C3B—C12B—N14B	137.7 (2)
C2—C3—C12—N14	−46.7 (3)	C2B—C3B—C12B—N14B	−45.5 (3)
N14—C12—N13—C21	0.4 (2)	C3B—C12B—N13B—C21B	−179.77 (19)
C3—C12—N13—C21	−179.16 (19)	C3B—C12B—N14B—C26B	179.69 (19)
N14—C12—N13—H14	−0.3	C12B—N13B—C21B—C26B	−0.3 (2)
C3—C12—N13—H14	−179.9	H14B—N13B—C21B—C26B	0.2
N13—C12—N14—C26	−0.5 (2)	C12B—N13B—C21B—C22B	179.2 (2)
C3—C12—N14—C26	179.07 (19)	H14B—N13B—C21B—C22B	179.7
C12—N13—C21—C26	−0.2 (2)	N13B—C21B—C22B—C23B	−180.0 (2)
C12—N13—C21—C22	178.9 (2)	C26B—C21B—C22B—C23B	−0.5 (3)
N13—C21—C22—C23	−179.6 (2)	C21B—C22B—C23B—C24B	1.0 (3)
C26—C21—C22—C23	−0.6 (3)	C22B—C23B—C24B—C25B	−0.4 (4)
C21—C22—C23—C24	1.3 (4)	C23B—C24B—C25B—C26B	−0.6 (4)
C22—C23—C24—C25	−0.6 (4)	C12B—N14B—C26B—C25B	−178.7 (2)
C23—C24—C25—C26	−0.8 (4)	C12B—N14B—C26B—C21B	0.4 (2)
C12—N14—C26—C25	−178.0 (2)	C24B—C25B—C26B—N14B	180.0 (2)
C12—N14—C26—C21	0.4 (2)	C24B—C25B—C26B—C21B	1.0 (3)
C24—C25—C26—N14	179.5 (2)	N13B—C21B—C26B—N14B	−0.1 (2)
C24—C25—C26—C21	1.4 (3)	C22B—C21B—C26B—N14B	−179.66 (19)
C22—C21—C26—N14	−179.28 (19)	N13B—C21B—C26B—C25B	179.1 (2)
N13—C21—C26—C25	178.4 (2)	C22B—C21B—C26B—C25B	−0.5 (3)
C22—C21—C26—C25	−0.7 (3)		

### Hydrogen-bond geometry (Å, °)

**Table:**

*D*—H···*A*	*D*—H	H···*A*	*D*···*A*	*D*—H···*A*
N14—H14···N13^i^	0.88	2.05 (1)	2.851 (2)	150
N14B—H14B···N13B^ii^	0.88	2.02 (1)	2.826 (2)	151

Symmetry codes: (i) *x*, −*y*+2, *z*−1/2; (ii) *x*, −*y*+1, *z*−1/2.
